# Exploring the social, emotional and behavioural development of preschool children: is Glasgow different?

**DOI:** 10.1186/s12939-014-0129-8

**Published:** 2015-01-17

**Authors:** Louise Marryat, Lucy Thompson, Helen Minnis, Philip Wilson

**Affiliations:** Institute of Health and Wellbeing, University of Glasgow, RHSC (Yorkhill), Glasgow, G3 8SJ UK; Centre for Rural Health, University of Aberdeen, Centre for Health Science, Old Perth Road, Inverness, IV2 3JH UK

**Keywords:** Child development, Child, Preschool, Poverty

## Abstract

**Background:**

Glasgow City has poorer adolescent and adult health outcomes in comparison to demographically similar cities in England and the rest of Scotland. Until now, little exploration of differences in child development between Glasgow and other areas has been made. The authors hypothesized that the poorer health outcomes and lifestyle behaviours of adults, coupled with relative economic deprivation, may impact on child social, emotional and behavioural development, compared with children from other parts of Scotland.

**Methods:**

Data from the Growing Up in Scotland national birth cohort study were used. Differences between Strengths and Difficulties Questionnaire (SDQ) scores and child and family characteristics of children living in the Greater Glasgow and Clyde (GGC) Health board vs. other health boards were examined. Logistic regression and linear regression models were fitted in order to explore independent associations between health board and SDQ raw and banded scores, respectively, whilst controlling for other contributing factors.

**Results:**

Children in GGC were demographically different from those in other areas of Scotland, being significantly more likely to live in the most deprived areas, yet no difference was found in relation to the mental health of preschool-aged children in GGC. Children in GGC had slightly *better* SDQ Conduct Problems scores once demographic factors were controlled for.

**Conclusions:**

At 46 months, there does not appear to be any difference in Glasgow with regards to social, emotional and behavioural development. Glaswegian children appear to have slightly fewer conduct problems at this age, once demographics are taken into account. A range of theories are put forward as to why no differences were found, including the inclusion of areas adjacent to Glasgow City in the analysis, sleeper effects, and rater bias.

## Background

**Figure 1:**
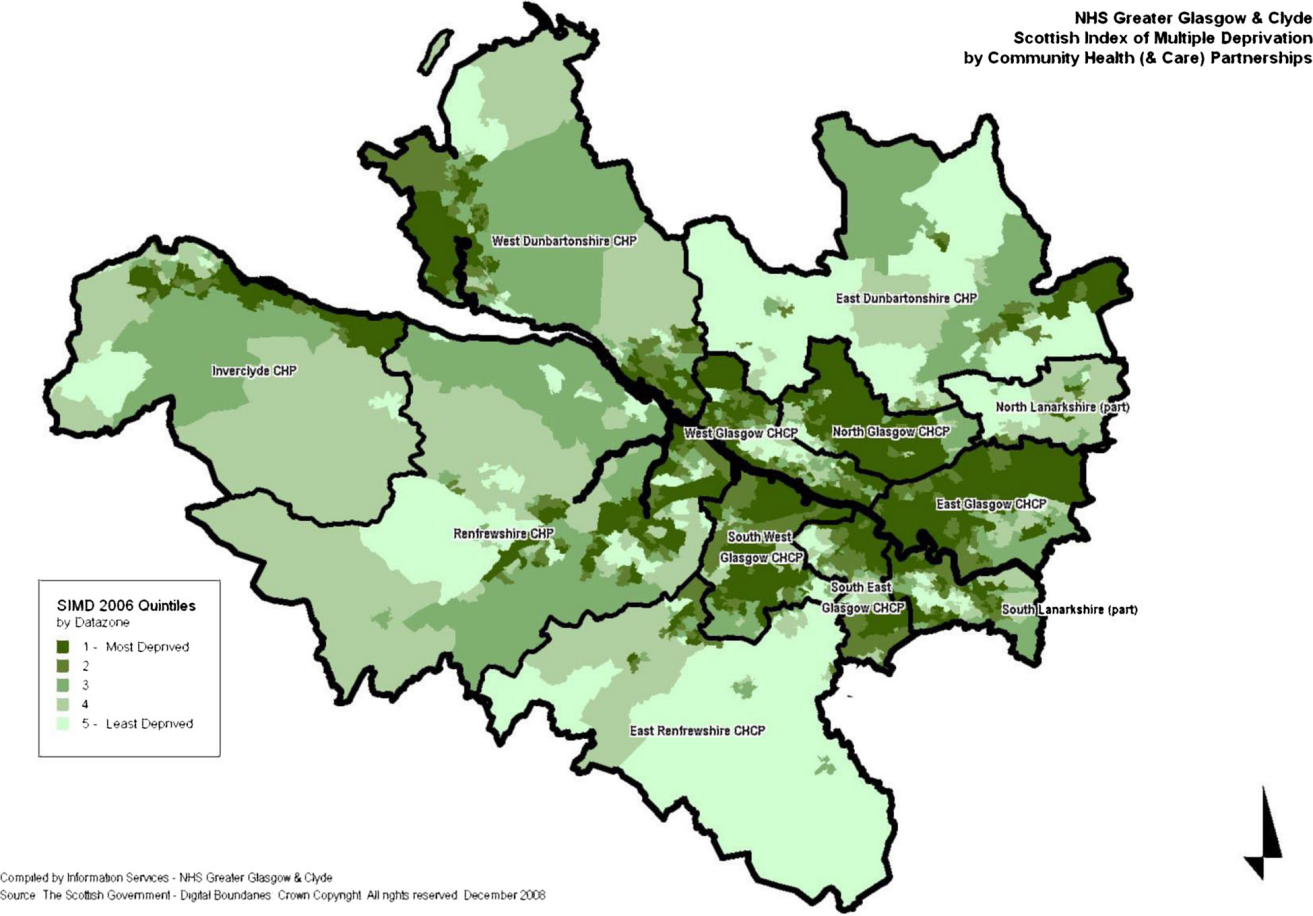
**Map of deprivation in greater glasgow and clyde health board.** This map reflects the boundaries and levels of area deprivation at the time of birth of the cohort children.

Glasgow is commonly known as ‘the sick man of Europe’, a title which relates to its poor health outcomes for adults, including increased premature mortality, anxiety, cardiovascular disease, general health and obesity [1-3]. In the past this has been attributed to poverty. Glasgow City has a high level of deprivation as Figure 1 demonstrates. Analysis of national area deprivation data indicates that of the 5% most deprived areas in Scotland, 52% were situated in Glasgow City in 2006 [4]. When Glasgow is compared to cities with almost identical demographic profiles, however, such as Manchester and Liverpool, the city has mortality rates which are 30% higher than these similar areas among working age adults [5]. Differences have been also been found between Glasgow City and other areas within Scotland in terms of diet, mental health, and physical health in adults, suggesting that there may be an additional difference in Glasgow City [6,7]. The majority of these differences can be explained by socio-demographic characteristics in Glasgow, however, some specific differences, for example, differences in intake of various foods, anxiety, heart-attacks, general health and overweight were not [6,8].

To date, there has been little research produced exploring whether Glasgow is different in relation to *children’s* health outcomes and the only published work, focusing on child mortality, has found no evidence of a difference in Glasgow [5]. Research on adolescent health outcomes demonstrated a decrease in self-reported physical health in Glasgow from the fourth year of secondary school, when young people are aged around 16, compared with the rest of Scotland, even once socio-demographic factors were controlled for. The same study found no differences in physical health earlier than this, or variations at any age on well-being, once adjusted to account for demographics, such as family affluence [7]. The, frequently unexplained, differences demonstrated in the current literature between Glasgow City and other areas in relation to adult health outcomes, combined with the lack of research which has been conducted around child outcomes, led to the current study’s exploration of whether Glasgow is different in terms of children’s outcomes, in particular an exploration of social, emotional and behavioural outcomes, which may demonstrate more variation in outcomes, compared with child mortality, for example.

Social, emotional and behavioural difficulties are important areas of study in themselves, as evidence indicates that they may have far reaching consequences, including future mental health problems and adverse adult outcomes, including increased levels of criminality and relationship problems, and poorer employment opportunities and fewer educational qualifications [9-11]. The preschool stage is a key time for children to develop skills which will allow them to engage fully in school life and to eventually be socially competent adults [12,13]. At this age in Scotland, all children are entitled to a minimum of 475 hours of free preschool education a year. This is taken up by 93% of families in Scotland [14], meaning that preschool may provide a good opportunity for an intervention to help develop social, emotional and behavioural competencies. It is important to understand if Glasgow City has higher levels of such difficulties in its young children and to understand the mechanisms behind this, in order to intervene early so that future problems may be prevented.

The preschool period in particular is an age where children spend much of their time in the home, and are heavily influenced by the behaviour of their parents. Several parenting practices in particular have been evidenced to contribute to children’s poor mental health: low levels of warmth, either through rejection or a lack of involvement, punitive or harsh discipline, including yelling, hitting and demands for obedience, and over-involved or over-protective parenting, including intrusion, encouraging dependence and the exclusion of outside influences [15,16]. Excessive levels of stress caused by sub-optimal parenting have been widely recognised to have a substantial effect on the development of children’s social emotional and behavioural functioning throughout childhood [17-20]. Furthermore, since the poorer adult mortality outcomes found in Glasgow are related to increased rates of deaths by suicide, drugs, alcohol and violence, it may be that there is an adverse effect of these parental behaviours seen in the prevalence of relatively young children’s social, emotional and, particularly, behavioural problems in Glasgow City [21,22].

The aim of this paper is to explore whether there is a difference in children’s social, emotional and behavioural problems at preschool age in the Greater Glasgow area, as reported by the child’s main carer, and whether any difference found can be explained by varying levels of deprivation in Glasgow and the rest of Scotland are controlled for. The current study uses data from the Growing Up in Scotland birth cohort to explore this phenomenon. We hypothesise that Glasgow City will have higher levels of social, emotional and behavioural difficulties in young children, in line with adolescent and adult health outcomes in the city, which cannot be explained purely by differences in deprivation levels.

## Methods

### The sample

This paper uses data from the Growing Up in Scotland Study (GUS). GUS is a national birth cohort study, which covers the whole of Scotland. The sample is stratified and clustered, and was derived from child benefit records which at the time of sampling included 97% of the population with children. Data zones (the key small-area geographic statistic in Scotland each containing 500–1000 people) were aggregated until each area had an average of 57 live births per year, based on the previous three years data, which was estimated to provide the required sample size. These Primary Sampling Units were then stratified by Local Authority (there are 32 geographically based Local Authorities in Scotland responsible for delivering local services) and then by Scottish Index of Multiple Deprivation Score, which is a measure of relative area level deprivation taking into account a range of local factors such as poverty, housing conditions, crime, employment [23]. Sweep 1 took place in 2005/6 when the children were 10 months old and began with 5,217 children [24]. The dataset used for this analysis comes from the fourth annual sweep of data collection, which took place when the children were 46 months old (in 2007/8), the time at which all children were eligible for a free pre-school place. By sweep 4 there were 3,394 children (65.1%) remaining in the sample.

Child social, emotional and behavioural outcomes are measured at Sweep 4 using the Parent-rated 4–16 year old version of Goodman’s Strengths and Difficulties Questionnaire (SDQ) [25]. This is a widely validated scale, frequently used in child cohort studies. It comprises 25 statements, which the informant marks as ‘Very true’, ‘Somewhat True’ or ‘Not at all true’ of the child in question. The statements break down into four negative scales: Conduct problems (e.g. often has temper tantrums), Hyperactivity/inattention (e.g. constantly fidgeting or squirming), Peer relationship problems (e.g. rather solitary/tends to play alone) and Emotional symptoms (e.g. has many fears/worries) and one positively rated scale – Prosocial behavior (e.g. helpful if someone is feeling upset, ill or hurt). The four negative scales can be combined into a Total Difficulties scale. Cut-offs are provided with the scale, creating a ‘normal’ and ‘abnormal’ grouping defined such that the ‘abnormal’ group should equate to 10% of the UK population. This analysis uses both the continuous and categorical scores. The SDQ has been found to have good predictive validity. Children who scored in the likely difficulties range of the SDQ (as rated by parents or teachers) had increased odds of 15 for being subsequently diagnosed with a psychiatric disorder 4–6 months later. Children who rated themselves as having likely difficulties on the self-complete version of the SDQ, had odds of a psychiatric diagnosis 6 times higher [26].

The GUS dataset contains a Health Board indicator, which includes NHS Greater Glasgow and Clyde health board (NHSGGC). Health Boards are organizations which are responsible for delivering state-funded healthcare in Scotland. There are 14 geographically based Health Boards in Scotland. There are 865 NHSGGC cases in the dataset and 3129 cases from other Scottish Health Boards. Ideally this analysis would look solely at Glasgow City, rather than the wider GGC Health board due to demographic differences between the two geographies, however small numbers at a city-level prohibit this. It is important to note therefore, that differences exist between the Glasgow City population Scottish Index of Multiple Deprivation (SIMD) patterns and the GGC SIMD patterns: half of the population of Glasgow City resides in the most deprived SIMD quintile areas, in contrast to about 36% of both the GGC population and GGC GUS sample. Furthermore, just 8.4% of the Glaswegian population live in the least deprived areas, compared with 18.6% of the GGC population as a whole and 21.9% of the GGC GUS sample [27]. It is also important to note that the sample was designed to be representative of the Scottish population and not the GGC population, which may also account for some differences in population estimates within the sample.

Demographic variables were chosen where there was previous evidence of a relationship with the outcome measures [28-32]. These were: ethnicity; sex; household banded income; family type; household socio-economic classification (NS-SEC); mother’s formal educational qualifications; age of mother at birth of cohort child; mother’s employment; level of area deprivation (SIMD); and area urban–rural classification.

### Analysis plan

We first assessed demographic differences between children living in NHS GGC and other Scottish health boards before going on to examine whether any differences were accounted for by variations in demographic profiles. Pearson correlations were performed to explore binary relationships between continuous SDQ scores, socio-demographic characteristics of the family and area in which the child lives, and Health Board. Binary scores were also explored as it was hypothesized that there may be differences between Health Boards in the proportions of children scoring in the ‘abnormal’ range on the scales, compared with the spread across the whole scale. Children’s SDQ scores were grouped into ‘normal’ and ‘abnormal’ scores, using the standard SDQ cut-offs [25]. Binary ‘abnormal’ and ‘normal’ SDQ scores were analysed and binary Spearman correlations examined between Health Board and SDQ means and ‘abnormal’ scores. Scores for children in Greater Glasgow and the rest of Scotland, respectively, were also compared with the UK norms for 5–10 year olds, using data from the survey of Mental Health of Children and Young People in Great Britain [31]. The data were weighted using the Birth Cohort Sweep 4 specific weight, which helps to control for both the differential response and attrition experienced in the survey.

Multivariate analysis was then carried out in order to assess if any difference between Glasgow and other areas in Scotland could be found once adjustments are applied for socio-demographic variables. The square roots of the continuous scores were used as the dependent variables, in an attempt to ‘normalise’ what would otherwise be heavily skewed data [33]. Results from Pearson correlations were analysed in order to ascertain co-linearity in the data (Table 1). No demographic variables had a correlation of r = 0.8 or higher, suggesting that co-linearity was not an issue in the data. Weighted forward stepwise linear regression models were fitted for the Total Difficulties scale and the individual sub-scales using the continuous scores, in order to examine whether differences were evident between GGC and the rest of Scotland in terms of where children fell on the total scale once other demographic variables were taken account of.

**Table 1:** **Pearson correlations between SDQ scores (continuous) and socio-demographic variables**

	**1.**	**2.**	**3.**	**4.**	**5.**	**6.**	**7.**	**8.**	**9.**	**10.**	**11.**	**12.**	**13.**	**14.**	**15.**
1. Total Difficulties	1														
2. Conduct	0.72**	1													
3. Hyperactivity	0.79**	0.48**	1												
4. Emotional	0.63**	0.20**	0.24**	1											
5. Peer problems	0.62**	0.26**	0.23**	0.37**	1										
6. Pro-social	−0.36**	−0.34**	−0.30**	−0.13**	−0.24**	1									
7. Sex of child	−0.12**	−0.08**	−0.15**	−0.02	−0.06**	0.14**	1								
8. Ethnicity (White)	0.08**	0.01	0.04*	0.05**	0.13**	−0.02	−0.01	1							
9. Mother education (No qualifications)	−0.23**	−0.18**	−0.17**	−0.16**	−0.13**	0.06**	0.01	−0.07**	1						
10. Household income (Lowest)	−0.25**	−0.20**	−0.17**	−0.16**	−0.17**	0.06**	0.03*	−0.11**	0.46**	1					
11. Mother’s employment	0.14**	0.11**	0.10**	0.10**	0.09**	−0.07**	−0.02	0.10**	−0.30**	−0.38**	1				
12. Household NSSEC (Managerial and Professional)	0.23**	0.19**	0.17**	0.15**	0.15**	−0.03*	−0.03	0.02	−0.52**	−0.58**	0.32**	1			
13. Age of mother at birth of child (20 or under)	−0.19**	−0.14**	−0.17**	−0.11**	−0.06**	−0.08**	0.03	−0.01	0.03	0.21**	−0.34**	−0.16**	1		
14. Urban/rural classification (Large urban)	−0.05**	−0.05**	−0.05**	−0.03	−0.02	−0.01	−0.01	−0.11**	0.10**	0.04*	0.01	−0.07*	0.07**	1	
15. SIMD (2006) (Least Deprived)	0.21**	0.17**	0.16**	0.13**	0.14**	−0.03	0.00	0.05**	−0.37**	−0.46 **	0.18**	0.43**	−0.32**	−0.20**	1
16. Health board (GGC)	−0.00	0.01	−0.01	0.01	−0.02	0.01	0.01	−0.17**	0.05*	0.01	−0.00	0.00	0.01	0.34**	−0.09**

*p<0.05, **p<0.01.

A series of models was then constructed for the binary SDQ banded scores. Co-linearity in the data was firstly explored through Spearman correlations, but all demographic correlations met the criteria for inclusion in the models (Table 2). These models explored relationships between being in the ‘abnormal’ range on each of the subscales, demographic factors and Health board, using a weighted forward stepwise logistic regression model. Following this model, any variables which were not significant in Model 1, or which had small numbers of cases, were removed and the remaining significant variables were entered into a weighted forward stepwise logistic regression model. The final model produced by this forward stepwise regression, along with the Health Board indicator, was then re-run as a forced entry model using the complex survey module, which takes account of the clustered and stratified nature of the sample.

**Table 2:** **Spearman correlations between banded SDQ scores (normal vs. abnormal) and socio-demographic variables**

	**1.**	**2.**	**3.**	**4.**	**5.**	**6.**	**7.**	**8.**	**9.**	**10.**	**11.**	**12.**	**13.**	**14.**	**15.**
1. Total Difficulties	1														
2. Conduct	0.45**	1													
3. Hyperactivity	0.41**	0.29**	1												
4. Emotional	0.35**	0.14**	0.10**	1											
5. Peer problems	0.42**	0.15**	0.10**	0.17**	1										
6. Pro-social	0.18**	0.14**	0.20**	0.06**	0.16**	1									
7. Sex of child	−0.07**	−0.04**	−0.09**	0.01	−0.04**	−0.05**	1								
8. Ethnicity (White)	0.08**	0.02	0.04**	0.02	0.07**	0.06**	−0.01	1							
9. Mother education (No qualifications)	−0.13**	−0.13**	−0.11**	−0.06**	−0.03	−0.05**	0.01	−0.05**	1						
10. Household income (Lowest)	−0.16**	−0.15**	−0.11**	−0.07**	−0.08**	−0.06**	0.03*	−0.12**	0.50**	1					
11. Mother’s employment	0.09**	0.09**	0.08**	0.05**	0.05**	0.05**	−0.02	0.10**	−0.28**	−0.40**	1				
12. Household NSSEC (Managerial and Professional)	0.15**	0.15**	0.10**	0.07**	0.06**	0.05**	−0.04*	0.04**	−0.55**	−0.60**	0.29**	1			
13. Age of mother at birth of child (Age 20 or under)	−0.09**	−0.10**	−0.12**	−0.05**	−0.02	−0.03*	0.03	−0.02	0.26**	0.34**	−0.15**	−0.38**	1		
14. Urban/rural classification (Large urban)	−0.02	−0.02	−0.04**	−0.01	−0.00	−0.04*	0.00	−0.14**	0.06**	0.02	0.00	−0.04**	0.05**	1	
15. SIMD (2006) (Least Deprived)	0.13**	0.14**	0.12**	0.04**	0.05**	0.04**	0.00	0.06**	−0.38**	−0.46**	0.16**	0.44**	−0.34**	−0.16**	1
16. Health board (GGC)	−0.08	0.01	−0.03*	0.01	−0.02	0.00	−0.00	−0.16**	0.02	−0.00	0.01	0.00	0.01	0.39**	−0.08**

*p<0.05, **p<0.01.

Due to the demographic differences between Glasgow City and the GGC Health Board, the last section of the analysis attempted to restrict the GGC sample as far as possible to Glasgow City, in order to establish if using the wider GGC variable was ‘diluting’ differences between the areas. This was done by restricting analysis to children living in Large Urban areas and in the most deprived quintile of the SIMD. These children were compared with children living in large urban areas out with GGC.

## Results

### Demographic characteristics of children in GGC and the rest of Scotland

**Figure 2:**
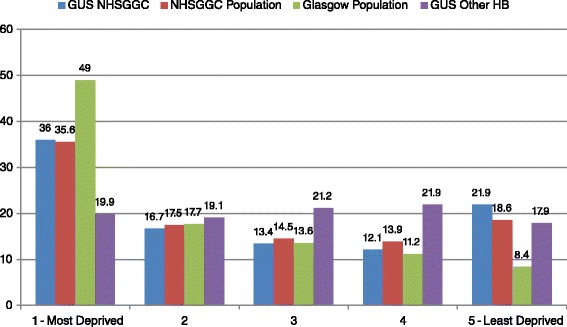
**Scottish index of multiple deprivation quintiles by area and sample.**

The Greater Glasgow and Clyde (GGC) sample of families in GUS appeared to significantly differ from the rest of the Scottish GUS sample demographically. The GGC participants were significantly more likely to live in an area of high deprivation than families from other Scottish health boards (36% in GGC, in contrast to 19.1% in other health boards) (Figure 2). Nevertheless, the proportion of families living in the *least* deprived areas in the GGC GUS sample is also slightly higher compared to other Scottish health boards (21.9% vs. 17.9%). When exploring household income in the weighted data, 29% of the GGC children lived in a household with an equivalised ^a^ household income in the lowest 20% of the sample population, whilst in other Health boards there was 24.3% (p < 0.01). Again, as with the area level deprivation data, GGC families were also slightly more likely to be in the highest income group, compared with families in other Scottish health boards (19.2% compared with 15.9%). Mirroring this was the socio-economic classification of families, with significantly larger proportions of GGC families being in the ‘Not Working’ group (7.1% vs. 3%), but also in the Managerial and Professional Group (37% vs. 33.6%).

Children in the GGC sample were significantly more likely to be from an ethnic minority background (9.6% in GGC compared with 2.1% in the rest of the sample). The GUS GGC participants were also significantly more likely to have a mother with no educational qualifications (13% compared with 7.5% in other health boards), though they were equally likely to have a mother with a degree level qualification or higher. The mother was also more likely to be working full time in GGC (58.1% vs. 54.6%), less likely to work part time (6% compared with 10.5%), and equally likely to not work (35.9% and 34.9%, respectively).

There were no statistically significant differences found between GGC families and other families in terms of the proportions of males, the proportions of lone parents or age of mother at the birth of the cohort child in the sample, which is somewhat surprising given how socially patterned these indicators usually are. This may indicate sample bias or differential attrition in the GGC sample.

### Levels of social, emotional and behavioural difficulties

Mean raw scores for the Total Difficulties scale and its respective subscales were assessed to see if there were any differences between children residing in GGC and those in other Health Boards. We found no difference between children from GGC and Other Health Boards on any of the SDQ subscales or on the Total Difficulties scale in relation to mean scores. In comparison to the UK norms, based on parent-rated data for 5–10 year olds in the British Mental Health Survey of Children and Young People [31], both GGC and other health boards in Scotland had slightly lower scores on Emotional Symptoms and the Total Difficulties scale, with slightly higher scores on the Conduct Problems scale. The GGC and Rest of Scotland samples from GUS had a mean of 8.0 on the Total Difficulties scale (standard deviation = 4.7), respectively, slightly lower than the mean of 8.6 for the UK sample. In contrast the mean for Conduct problems in GGC and the rest of Scotland in the GUS sample was 2.0 (standard deviation = 1.5), respectively, compared with a mean of 1.6 in the UK sample (Table 3).

**Table 3:** **Means for SDQ subscales and total difficulties for GUS GGC, GUS other health boards and UK norms**

	**Greater glasgow and clyde – mean (SD)**	**Other health boards mean (SD)**	**UK norms (5–10 year olds)** **[** 31 **]**
**Emotional symptoms**	1.2 (1.4)	1.2 (1.4)	1.9 (2.0)
**Conduct problems**	2.0 (1.5)	2.0 (1.4)	1.6 (1.7)
**Hyperactivity/Inattention**	3.7 (2.3)	3.7 (2.2)	3.6 (2.7)
**Peer problems**	1.2 (1.5)	1.2 (1.4)	1.4 (1.7)
**Total difficulties**	8.0 (4.7)	8.0 (4.5)	8.6 (5.7)
**Pro-social**	7.8 (1.7)	7.9 (1.8)	8.6 (1.6)
**Bases**	919 – 925	3016 – 3044	5855

**Figure 3:**
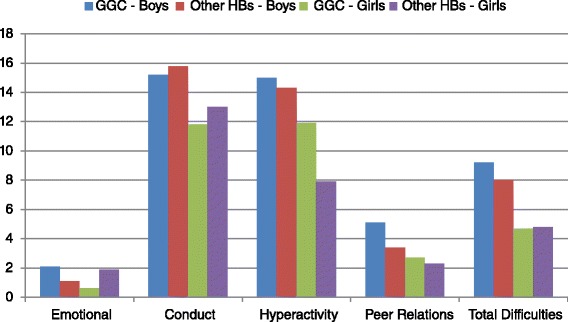
**Proportions of children with abnormal scores on each subscale by health board and sex.**

When levels of children in the ‘abnormal’ range of the scales were explored, no statistically significant differences were found. Children in GGC actually had slightly lower levels of conduct problems in the ‘abnormal’ range (13.4% in GGC vs. 14.4% in other Scottish Health Boards), despite having a higher mean score, though this was not significant. A higher proportion of GGC children were in the ‘abnormal’ range on the Hyperactivity/inattention scale (13.4% vs. 11.1%), and, to a lesser extent, in the areas of Peer Relations (3.9% vs. 2.9%) and Total difficulties (7% vs. 6.5%). ‘Abnormal’ Emotional difficulties scores are rare at this age [34] and little difference can be seen between areas (3.1% of GGC children with an ‘abnormal’ score compared with 3.3% in other Health boards) (Figure 3).

### What variables are associated with higher levels of difficulties at preschool?

Pearson correlations were performed using the continuous scores to analyse significant associations between SDQ scores, Health Board and demographic characteristics of the children and their families. Living in the GGC Health Board showed no significant unadjusted correlation with any continuous SDQ score. The GGC population of families in GUS did appear to differ demographically from the rest of the Scottish GUS sample however. Pearson correlations using continuous scores showed GGC children differed from those in other Health Boards in terms of the lower educational qualifications of the mother, the increased number of children from Ethnic Minorities in the population, the higher level of area deprivation and the greater proportion of large urban areas within the Health Boards. Aside from the negative correlation between being in a rural area and living in GGC (r = −0.34), correlations for demographic differences between areas were small. In terms of demographic correlations with SDQ continuous scores, a wide variety of demographic factors, such as the sex of the child, household income and maternal education, were demonstrated to have significant correlations with the various scales (see Table 1).

As with the treatment of continuous scores, Spearman correlations were used to examine unadjusted significant correlations between binary SDQ groups and both Health board and demographic characteristics of the children and their families. There was a very small but significant correlation between being in the ‘abnormal’ Hyperactivity/Inattention group and Health board, with children living outwith GGC being less likely to have an ‘abnormal’ hyperactivity/inattention score. As with the continuous scores, a wide range of demographic factors was associated with being in the various ‘abnormal’ difficulties groups (Table 2).

### Multivariate analysis

Linear regression models were fitted in relation to the continuous Total Difficulties scores and its four constituent sub-scales in order to assess whether differences were present between GGC and other Health Boards when adjusted for demographic profiles of the area’s samples. Controlling for the difference in demographics, it was evident that living in the GGC area was independently associated with one sub-scale – Conduct Problems. However, this was a negative association (β = −0.14), meaning that living in the Greater Glasgow and Clyde area was significantly associated with a lower Conduct Problems score, when compared with living in another Scottish health board, once levels of deprivation etc. were taken account of. The models only explained 4% of the variation on the Emotional Symptoms scale to 12% on the Total Difficulties scale (Table 4).

**Table 4:** **Linear regression model of correlations with higher difficulties scores on total difficulties and each sub-scale**

	**Total difficulties**	**Emotional**	**Hyperactivity**	**Conduct**	**Peer relations**
**Health board**	NS	NS	NS	*	NS
Glasgow	−0.37	−0.07	−0.04	−0.14	−0.05
Other	-	-	-	-	
**Multiple deprivation (SIMD)**	**	NS	*	**	**
5 – Least deprived	−1.00		−0.29	−0.31	−0.33
4	−1.15		−0.42	0.31	−0.30
3	−0.68		−0.16	−0.15	−0.25
2	−0.50		−0.13	−0.18	−0.17
1 – Most deprived	-		-	-	-
**Ethnicity**	*	NS	NS	NS	**
White	−1.26				−0.81
Non-White	-				-
**Mother’s education**	**	**	**	**	NS
No qualifications	1.59	0.42	0.56	0.43	
Other	1.84	0.98	0.48	0.52	
Lower standard grade or equivalent	1.70	0.48	0.76	0.32	
Higher standard grades or equivalent	0.93	0.17	0.54	0.25	
Higher grades or equivalent	0.01	−0.01	0.14	−0.04	
Degree or higher	-	-	-	-	
**Equivalised income**	**	**	*	**	**
Bottom Quintile (<£11, 875)	1.48	0.39	0.43	0.42	0.46
2^nd^ Quintile	0.88	0.27	0.19	0.22	0.39
3^rd^ Quintile	0.44	0.05	0.17	0.07	0.18
4^th^ Quintile	0.19	−0.18	0.05	0.08	0.08
Top Quintile (> = £37,500)	-	-	-	-	-
**Household employment status**	NS	NS	NS	NS	NS
1+ Parent Work Full-time (16+ hours)					
1+ Parent Work Part-time (<16 hours)					
No work					
**Household socio-economic classification (NS-SEC)**	NS	NS	NS	NS	NS
Managerial and Professional					
Intermediate					
Small employers and own accounts workers					
Lower supervisory and Technical					
Semi-routine and routine					
Never worked					
**Child sex**	**	NS	**	**	**
Male	1.05		0.63	0.22	0.16
Female	-		-	-	-
**Age of mother at birth of child**	**	NS	**	**	NS
20 years or under	1.88		1.07	0.46	
21 to 30 years	0.93		0.73	0.18	
31 to 40 years	0.52		0.50	0.15	
Over 40 years	-		-	-	
**Family status**	NS	NS	NS	NS	NS
Couple Family					
Lone Parent					
*R* ^*2*^ *(Nagelkerke)*	0.12	0.04	0.08	0.07	0.05
*Base*	3695	3769	3740	3746	3768

*p<0.05, **p<0.01.

Logistic regression models were then fitted in order to investigate whether there were any significant differences in terms of the levels of social, emotional and behavioural difficulties classified as ‘abnormal’ for children living in the GGC health board, once demographics were controlled for. Living in GGC was found to be not independently correlated with any type of social, emotional or behavioural ‘abnormal’ score, once demographics such as area deprivation were taken account of. Being in the ‘abnormal’ range of the SDQ scales was associated with a range of demographic characteristics. Children were more likely to fall into the ‘abnormal’ range of the Total Difficulties score, for example, if they were male (β = 0.64), had a non-White UK ethnicity (β = −0.70 for White children compared with non-White), had a mother with lower educational qualifications (β = 0.91 for mothers with no qualifications compared with those with a Degree or higher) and had a lower household equivalised income (i.e. adjusted for the number of people in the household) (β = 1.64 for those in the bottom quintile compared with the top). This model explained the largest proportion of the variation in terms of ‘abnormal’ scores, though this was still only 11% (Table 5).

**Table 5:** **Predictors of abnormal scores on the SDQ total difficulties scale and subscales by child, family and area characteristics**

	**Total difficulties**	**Emotional**	**Hyperactivity**	**Conduct**	**Peer relations**
**Health board**	NS	NS	NS	NS	NS
Glasgow	−0.10	−0.13	0.15	−0.24	0.17
Other	-	-	-	-	-
**Multiple deprivation (SIMD)**	NS	NS	**	**	NS
5 – Least deprived			−0.51	−0.68	
4			−0.54	−0.60	
3			−0.27	−0.31	
2			−0.18	−0.25	
1 – Most deprived			-	-	
**Ethnicity**	*	NS	NS	NS	*
White	−0.70				−0.97
Non-White	-				-
**Mother’s education**	**	**	**	NS	NS
No qualifications	0.91	1.15	0.31		
Other	0.85	2.62	0.38		
Lower standard grade or equivalent	0.80	0.26	0.56		
Higher standard grades or equivalent	0.48	0.87	0.47		
Higher grades or equivalent	−0.05	−0.21	0.01		
Degree or higher	-	-	-		
**Equivalised income**	**	NS	NS	**	**
Bottom Quintile (<£11, 875)	1.64			0.94	1.02
2^nd^ Quintile	1.16			0.44	0.97
3^rd^ Quintile	0.51			0.24	−0.05
4^th^ Quintile	0.68			0.02	−0.07
Top Quintile (> = £37,500)	-			-	-
**Household employment status**	NS	NS	**	NS	NS
1+ Parent Work Full-time (16+ hours)			−0.32		
1+ Parent Work Part-time (<16 hours)			−0.46		
No work			-		
**Household socio-economic classification (NS-SEC)**	NS	NS	NS	NS	NS
Managerial and professional					
Intermediate					
Small employers and own accounts workers					
Lower supervisory and technical					
Semi-routine and routine					
Never worked					
**Child sex**	**	NS	*	**	*
Male	0.64		0.54	0.23	0.47
Female	-		-	-	-
**Age of mother at birth of child**	NS	NS	**	NS	NS
20 years or under			1.37		
21 to 30 years			1.10		
31 to 40 years			0.76		
Over 40 years			-		
**Family status**	NS	NS	NS	NS	NS
Couple family					
Lone parent					
*R* ^*2*^ *(Nagelkerke)*	0.11	0.05	0.08	0.06	0.05
*Base*	3716	3739	3924	3776	3768

*p<0.05, ** p<0.01.

**Figure 4:**
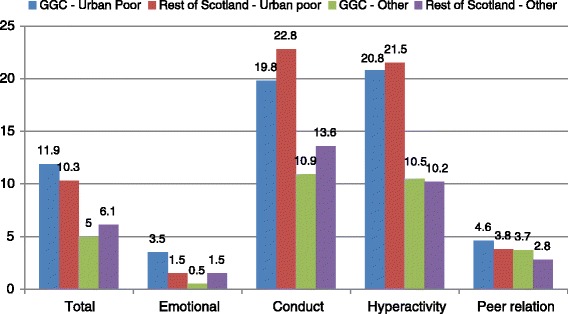
**Proportion of children in the abnormal range of each scale by GGC and rest of Scotland by urban-deprivation status.**

In order to explore whether the differences seen in Glasgow children’s outcomes were simply an artifact of Glasgow City being both a large urban area and very deprived, the analysis was then restricted to just children who lived in a Large Urban Area, as classified by the Scottish Government, and to children who lived in an area in the most deprived quintile on the Scottish Index of Multiple Deprivation [35]. This therefore excluded most of the surrounding areas of Glasgow City, with the exception of some large towns, such as Renfrew, which arguably share characteristics with Glasgow City. On all scales with the exception of Emotional Symptoms, living in a Large Urban area in the most deprived area Quintile, regardless of Health Board, was associated with a substantial increase in the proportion of children with ‘abnormal’ scores: for example, 11.9% of GGC urban-poor children and 10.3% of other Health Board urban-poor scored in the ‘abnormal’ range of the Total Difficulties scale, compared with 5% of non-urban poor GGC children and 6.1% of other Health Board non-Urban-poor, suggesting that it is both living in a large Scottish city and experiencing poverty that affects children’s development, both in Glasgow City and elsewhere. The Emotional Symptoms ‘abnormal’ scores were the exception to this. Although proportions of children scoring in the ‘abnormal’ range are very low across the board, children in GGC Urban-poor areas appear to be more likely to have an ‘abnormal’ score (3.5% ‘abnormal’) compared with all other children, including those from Urban-Poor areas in the rest of Scotland (1.5%), however numbers, particularly in the GGC sample, were very small and differences were not significant (Figure 4).

## Discussion

This paper aimed to explore whether there was a difference between Glasgow and other areas in Scotland in the prevalence of social, emotional and behavioural difficulties in preschool aged children, and, in particular, whether there is an amount of variation between the areas which cannot be explained solely by differences in the demographic profile of Greater Glasgow and Clyde. Results indicate that there appears to be a small association between living in the GGC area and SDQ scores, though only on the continuous SDQ Conduct Problems scale. Contrary to expectation, this appears to be a negative association, whereby continuous Conduct Problem scores in GGC are actually slightly better than those in the rest of Scotland, once demographic characteristics of the family and area are taken account of. Furthermore, when exploring the adjusted ‘abnormal’ and ‘normal’ SDQ scores and all other continuous SDQ scores, GGC preschoolers do not differ from the those in the rest of Scotland. It would appear, therefore, that the differences in the unadjusted proportions of children scoring in the ‘abnormal’ range in GGC, particularly in terms of Conduct Problems, Hyperactivity/inattention and Peer Relationship Problems, are accounted for by the differences which can be seen in the demographic profiles of the two samples i.e. the differences in levels of deprivation, education levels of mothers and ethnicity explain all of the variance between the GGC and other areas.

The lack of other differences between GGC and other Scottish Health boards (both in terms of continuous and banded scores) raises questions about whether there really is no difference present during childhood for Glasgow’s children, or whether any difference is masked by the sample in the Growing Up in Scotland Study. In line with the little evidence there is in this area, it could be that there is no difference seen in Glasgow at this age in terms of child social, emotional and behavioural difficulties [7]. Another theory is that the differences in the early experiences of children in Glasgow, such as witnessing more violence and experiencing greater deprivation, may have an impact, but that this may be a ‘sleeper effect’ i.e. that the impact of these experiences may not be seen until later in childhood. Indeed, the only previous work in this area of development indicated that possible mental health difficulties emerged at age sixteen [36]. There has been considerable debate about sleeper effects in child development in recent years, with some academics now disputing their existence [37]. For others though there is a view that early adverse experiences may lie dormant for years before materializing as mental health issues, violence or delinquency [38,39]. If the latter view is correct, it could be that these early adversities play a role in the poorer adult outcomes in Glasgow, which can be seen in terms of the excess premature mortality through violence, drug and alcohol misuse and suicide, particularly in the male population [40]. A recent tentative finding suggested that children in Glasgow City may be more likely to have witnessed domestic violence than their counter-parts in other, demographically similar, cities [41]. The impact of witnessing domestic violence on children’s emotional development has been well-documented [42-44].

However, the lack of a difference between Glasgow and other parts of Scotland, once demographics are controlled for, could also be an artifact of the sampling frame. The GUS sample was demonstrated in the analysis to be different to that of the population of GGC, in that it was both more affluent and more deprived at the extremes of each end of the scale. The fact that this is a cohort sample which, as with the majority of cohort studies, suffers from differential attrition across the years, may exacerbate this sampling issue [45]. GUS disproportionately loses the most vulnerable families e.g. those with younger mothers and those from more deprived areas, however, these characteristics are, on the whole, accounted for by the survey weighting. Evidence from other cohort studies though suggests that there may be selective attrition from families containing children with more behavioural problems, which would not be picked up in the weight over and above deprivation [45].

Furthermore, GUS is only able to explore differences at a Health Board level, due to small numbers at the city level. Previous differential outcomes in the literature have primarily been present when looking at Glasgow City alone. As the analysis showed, the population of GGC is substantially different to Glasgow City in terms of its demographic characteristics. Furthermore, in the mid-Twentieth Century, families were cleared out of the slum areas of Glasgow City in the Glasgow City over-spill scheme. Movement was not uniform: more highly skilled workers and those from higher social categories were moved out of Glasgow City into the surrounding suburban and rural areas of GGC, whereas unskilled and lower social class families were re-housed in the North East and South of the city [46]. The addition of Glasgow City’s more affluent and potentially more highly educated neighbours from surrounding areas to form the GGC Health Board for analysis may mean that any unexplained variation in Glasgow City has been diluted by the effects of housing policy.

In an attempt to isolate the GUS GGC sample to Glasgow City as far as possible, our analysis was split by families living in both Large Urban areas and in the most deprived area quintile, and all others. Using these criteria, GGC and Other Health Boards were shown to both have fairly similar levels of difficulties, with substantially higher levels of ‘abnormal’ scores seen for families in all areas living in Urban-Poor areas compared with other areas. The analysis therefore indicates that the difference in adult outcomes in Glasgow City, could be a result of living in a Large Urban Deprived area of Scotland. Studies exploring such differences have looked at Glasgow City versus the rest of Scotland and Glasgow City versus demographically similar cities in England (e.g. [2,5,8,47]). However, until now, no studies have compared Large Urban Deprived areas within Scotland. It may be that there is something systematically different about these areas in Scotland in comparison with their counterparts in England, for example the geographical distance between some urban centres in Scotland may have an isolating effect on people living within these areas which may lead to poorer outcomes. Further analysis using larger datasets is required in this area.

Finally, this survey uses parent-rated SDQs. If parents are used to seeing different levels of problematic behaviours in different areas then their view of ‘normality’ may be very different. If parents view their child’s aggressive behavior, for example, as ‘normal’ in relation to his or her peers, they may give lower scores than parents living in areas with less aggression. It is therefore possible that children in GGC do have poorer social and emotional functioning, but this is not expressed by their parents. This may explain the unexpected finding that children in GGC had lower Conduct Problems scores than their peers in order areas. Social desirability, i.e. the wish to present oneself or one’s family in a desirable light [48], is another potential issue in survey research, particularly with parents. It may be that some parents would like to portray their child’s behaviour more positively than may be the reality, particularly in front of an interviewer. GUS attempts to minimize the impact of this through the parent/carer completing the SDQ in a self-completion module on a laptop, so that their answers are kept private from the interviewer. Results on the effect of mode of questionnaire delivery on social desirability are mixed however, with some studies finding under-reporting in face-to-face administered questionnaires, compared to self-complete, with others finding no difference between the two methods [49]. It is unclear why social desirability should have a greater impact in GGC than in other areas, although there is some evidence that more vulnerable families are more likely to attempt to give a favorable impression due to the fear of third party involvement [48]. The study is also limited by its reliance on a single rater. Previous research has found the using multiple informants provides a more accurate assessment of a child’s level of difficulties [50]. The use of teacher-rated data would help to address some of these problems, however, this was not available for Scottish children at the time of writing.

Of the remaining significant variables in the model, it was interesting to note that only child and family level characteristics had an independent relationship with child outcomes at this age. Previous research has reported that individual and household level characteristics have a stronger impact than area level factors in early to middle childhood [51,52]. It has been suggested that this is due to children spending more time around the home and with the primary carer at this stage [53]. There may also be a genetic effect, as evidenced in some previous research. The impact of these individual, parental and household factors is in line with Bronfenbrenner’s Ecological Systems theory, which posits that a range of factors work together at different levels to influence child development [54]. It may be that some children are more susceptible than others to these environmental influences and thus impact may not be uniform [55]. The identification of risk factors however may help to identify children who may need additional support to reach their optimal development.

In terms of child characteristics which were significant in the model, the relationship between being male and the greater likelihood of experiencing social, emotional and, particularly, behavioural problems and hyperactivity, was strong here. This relationship has been well-evidenced in previous research across childhood [56].

In relation to family characteristics, maternal education is also closely related to poverty, and to parenting practices, which may affect child outcomes. Results from this analysis showed a gradient effect of decreasing odds in line with increasing educational qualifications from Standard Grades (exams taken at age 16 in Scotland) up to Degree level or higher. Mothers with Higher Grades or a Degree, had odds of having a child in the ‘abnormal’ Total Difficulties Group one quarter of those of a mother with no qualifications (i.e. having no Standard Grade qualifications). Previous research from the Growing Up in Scotland study has found that having no qualifications was correlated with a view that smacking or shouting at a child was very or fairly useful at age 34 months [57,58]. Harsh parenting, in turn, has a detrimental effect on social, emotional and behavioural outcomes in childhood (and beyond) [28,59,60].

At a household level, living in the most economically deprived households was clearly related to having more social, emotional and behavioural difficulties at 46 months, in comparison to those in all other income quintiles, but particularly those in the most affluent households, in similarity to previous research [56,61,62]. It is likely that this impact of living in poverty is not direct, but rather is mediated through a number of pathways. For example, increased parental stress through living in difficult circumstances can impact on poor or harsh parenting, which in turn may lead to poorer social, emotional and behavioural outcomes [52,63]. Furthermore the stress of poverty leaves parents with fewer psychological resources available to create a warm environment in the home, which may result in conduct issues and a lack of emotional regulation in these young children [52,64]. There may also be an additional intergenerational impact of poverty on maternal stress and child development, through the continuing cycle of poverty (for a review see Harper and colleagues) [65]. Further evidence demonstrates associations between parental substance misuse, the witnessing of violence and parental mental health problems on adverse child outcomes [43,66-69].

## Conclusions

No differences were found in children’s social, emotional or behavioural difficulties between Glasgow and other parts of Scotland in the current analysis. The only difference between children living in Greater Glasgow and Clyde’s levels of difficulties and other children’s, was slightly lower scores on the Conduct Problems domain, after adjustment for other demographic factors. A wide range of theories was put forward as to why no differences were found. One theory is that differences do not materialise until adolescence: it is questionable whether this is because of variations in experiences during adolescence, or whether a ‘sleeper effect’ is at work. It could be that the lack of differences found is related to the particular sample used during analysis: the GUS GGC sample is both more affluent and more deprived than the GGC population and, as a cohort, suffers from differential attrition, which may mean that children with difficulties may be more likely to drop out. Furthermore, the analysis was limited to examining Greater Glasgow and Clyde versus other Health Boards due to insufficient numbers in Glasgow City: it may be that using this wider sample masks any effect of living in Glasgow City itself. Finally, the fact that parents were rating the SDQs may introduce forms of bias particular to an area, again diluting the results. Further research is required which looks at the effects of living in Glasgow City, specifically, in relation to social, emotional and behavioural difficulties throughout childhood, in order to establish whether and when differences between Glasgow City and other parts of Scotland emerge during childhood. In addition, further exploration of the impact of living in large urban areas in Scotland on social, emotional and behavioural development would also be beneficial.

## Endnote

^a^Equivalised Household Income is when the total income is adjusted for the number of adults and the number of children of different ages in the household. The equivalised income quintiles are based on unweighted data.
